# Hypertension and associated factors in HIV-infected patients receiving antiretroviral treatment in Burundi: a cross-sectional study

**DOI:** 10.1038/s41598-022-24997-7

**Published:** 2022-11-28

**Authors:** Déo Harimenshi, Théodore Niyongabo, Pierre-Marie Preux, Victor Aboyans, Ileana Desormais

**Affiliations:** 1 Inserm U1094, IRD U270, CHU Limoges, EpiMaCT-Epidemiology of Chronic Diseases in Tropical Zone, Institute of Epidemiology and Tropical Neurology, OmegaHealth, University of Limoges, Limoges, France; 2 Department of Internal Medicine, CHU Kamenge, University of Burundi, Bujumbura, Burundi; 3 Department of Cardiology, CHU Limoges, Limoges, France; 4 Department of Vascular Surgery and Vascular Medicine, CHU Limoges, Limoges, France

**Keywords:** Cardiology, Risk factors

## Abstract

Currently, the life expectancy of people living with the human immunodeficiency virus (HIV) and the general population are similar. Hypertension is a major public health issue in Africa and is largely underdiagnosed. Most HIV-infected individuals, especially those on Anti-Retroviral Therapy (ART) have hypertension. Our project aims to determine the prevalence of hypertension and associated factors amongst HIV-infected adults treated by ART in Burundi. A cross-sectional study was conducted among HIV-infected subjects over the age of 20, managed in five healthcare centers for people living with HIV (PLWH). The World Health Organization STEPWISE survey and anthropometric measurements were employed. Blood pressure was measured according to the ESC 2018 recommendations. 1 250 HIV-infected patients aged between 35.4 and 50.2 years were included (18.4% men). The prevalence of hypertension was 17.4% (95% CI 13.2–22.1). Approximately 47.25% of HIV patients with hypertension were previously undiagnosed. Other factors were associated with HTN, such as being overweight (OR 2.88; 95% CI 1.46–5.62), obesity (OR 2.65; 95% CI 1.27–5.55), longer duration of HIV infection: ≥ 10 years (OR 1.04; 95% CI 1.14–3.20), diabetes (OR 2.1; 95% CI 1.37–3.32) and age (OR 1.13; 95% CI 1.09–1.14). Despite their young age, almost 20% of HIV-ART treated patients had hypertension, 50% of these were undiagnosed. Blood pressure monitoring is crucial in these patients, especially those identified as high-risk, with prompt life and disability-saving interventions.

## Introduction

Globally, 37.6 million people are living with HIV. 67% of these reside in sub-Saharan Africa^1^. Antiretroviral therapy (ART) protocols have enabled substantial decreases in HIV-related mortality and improved long-term survival.

Cardiovascular disease (CVD) is still the leading cause of premature death and morbidity^2^. In 2019, CVD killed approximately 17.9 million people, i.e., 32% of global deaths, 75% of these in developing countries. In addition to “classic” risk factors, such as smoking, alcohol consumption, and inactivity^3–6^, HIV-infected patients may have additional risk factors related to endothelial dysfunction and the metabolic effects of antiretroviral drugs such as dyslipidemias or insulin resistance. The chronic inflammation induced by Human Immunodeficiency Virus (HIV) can heighten HIV-infected patients predisposition to CVDs^7,8^. Previous studies reported that the prevalence of cardiovascular risk factors is often higher in HIV-infected patients than in the general population^9^. Around 24% of people (or 8.9 million) living with HIV have high blood pressure. These estimations vary according to the region and country income. The prevalence of hypertension in people living with HIV is significantly higher in North America and lower in sub-Saharan Africa and Asia, with no relevant differences between South American and European populations^10^. Hypertension is a public health issue in Africa, as largely under diagnosed^11,12^. Sub-Saharan Africa presented the highest prevalence of hypertension^13,14^. The WHO estimated that the prevalence of hypertension was very high in Africa, with nearly 46% of early adults (25 years old) or older being hypertensive.

Heterogenous studies suggested that HIV-infected people on ART have a higher prevalence of HTN than non-infected individuals^15–18^. In Kenya, a recent study showed a low prevalence of hypertension in HIV-negative individuals^19^. In general, the prevalence of hypertension in people living with HIV (PLWH) on ART and the ART-naive PLWH ranged from 6 to 50% and 2 to 41%, respectively^7^ .

Another larger meta-analysis including all studies worldwide showed that 35% of all HIV-infected adults on ART have HTN compared to healthy adults (an estimated 12%). Previous research highlighted that Systemic inflammatory processes, including the activation of the innate and adaptive immune systems, contribute to the development of hypertension in the population and in experimental animals. Therefore, the correlation between innate and adaptive immune factors and hypertension among HIV-infected individuals is still very unclear.

In Burundi, 82,000 people are living with HIV and 75 059 have received antiretroviral therapy, approximately 9356 are newly infected, with annual AIDS-related deaths of 2 300. At the end of 2021, screening and treatment of HIV-infected people was being carried out in 1 035 HIV-centers across the country^23^.

According to data from the Demographic and Health Surveys III 2016–2017, HIV infection in Burundi is “epidemic” with a prevalence of 0.9% among the general population aged 15 to 49 years with a seroprevalence of 1.2% in women and 0.6% in men of the same age^24^. Although the level of prevalence in the general population is lower, huge disparities can be observed and HIV affects certain categories severely, particularly key populations with prevalence varying between 4.8% among men who have sex with men and 21.3% among sex workers. The prevalence of HTN in PLWH in Burundi is unknown.

Consequently, our study aimed to determine the prevalence of HTN and associated factors in a large group of rural and urban HIV-infected adults on ART in Burundi.

## Methods

From December 2020 to October 2021, a cross-sectional study was conducted among all HIV-infected outpatients attending, consecutively, the five healthcare centers in five regions covering rural and urban areas in Burundi. All five centers were randomly assigned among all health care centers in each region. Inclusion criteria: age ≥ 20 years, positive HIV status and currently under ART (≥ 1 years). The exclusion criteria were age under 20, no ART or refusal to participate. The study obtained the approval of the National Ethics review Committee (NEC) of Burundi [Comité National d’Ethique pour la protection des êtres humains sujets de la recherche biomedicale et comportementale, Burundi (FM/CE/01/11/2020)]. For those who accepted to participate in our project, the free and informed consent of all subjects was obtained. The reported research was undertaken in compliance with the Declaration of Helsinki.

### Definitions and data collection

Systolic and diastolic blood pressure were measured in the sitting position, after a resting period of at least 15 min using an automatic device (OMRON M3, OMRON Corporation Japan). Three measurements were taken in both arms at 5 min intervals, in accordance with the 2018 ESC guidelines^25^. The mean of the last two measurements was used in the analyses. Hypertension was defined according to self-reported ongoing antihypertensive treatment, or SBP ≥ 140 mmHg and/or DBP ≥ 90 mmHg. Weight was measured without shoes and in light clothing using a Medisina^®^ scale to the nearest 100 g (Seca, Humburg, Germany), height was measured using a stadiometer without shoes to the nearest 0.1 cm. We calculated the body mass index (BMI) as: weight (kg)/height (m^2^).

BMI was classified as underweight BMI < 18.5 kg/m^2^, normal weight: BMI = 18.5–24.9 kg/m^2^, (BMI < 25 kg/m^2^), overweight (25–29.9 kg/m^2^ and obese (BMI ≥ 30 kg/m^2^). Adequate physical activity was defined according to the WHO 2020 guidelines^26^ as engaging in at least 150 min of moderate work or sports or at least 75 min of vigorous intensity work or sport per week.

The participants were considered smokers if they smoked at least one cigarette or other local tobacco product in the last 24 months. Alcohol use was assessed based on the frequency and amount of alcohol intake in a typical drinking week, and was categorized as never, occasional (less than 5 days/week) and regular (more than 5 days/week). Diabetes was defined if fasting blood glucose was 126 mg/dL (7 mmol/L)^27^ or higher or if under current antidiabetic treatment. Clinical information, such as duration of HIV infection, duration on ART, HIV clinical stages and types of ART regimens were recorded.

According to current Burundian viral load guidelines^28^, < 1000 copies/mL is defined as viral suppression, while viral load < 50 copies/mL is defined as undetectable. We used an adapted standardized WHO STEPS questionnaire to collect data that was then compiled during a face-to-face interview. A clinical examination and laboratory tests were conducted. Clinical data were extracted from Center records.

### Statistical analysis

Data forms were validated, coded, entered into the computer, and checked for quality control. The quantitative variables were presented using mean ± standard deviation (SD). The normality of continuous variables was analyzed by the Shapiro–Wilk test. The distribution of quantitative variables was compared by the Student t-test for variables following normal distribution and the Man Whitney test for variables not following normal distribution.

Qualitative variables were described as numbers, proportions, and quantitative variables as mean ± standard deviation (SD). Chi-square tests or Fisher’s exact test were used to compare proportions between groups. In order to determine independent risk factors associated with hypertension among HIV infected patients, a multivariate logistic regression model with backward stepwise procedure included was performed. Variables included in the final multivariate model were those with a p-value ≤ 0.25 in the univariate model. The level of significance for all the statistical analyses was set at 0.05. Data were analyzed using STATA 12 software packages (version 12.0, College Station, TX).

## Results

A total of 1 250 HIV infected patients took part in the study, 230 (18.4%) were men while 1 020 (81.6%) were women. There was no refusal to participate. The mean age of our participants was 42.8 ± 7.4 years. Almost 70% of our participants were from a rural background. Regarding marital status, 65.6% were married, 20.3% divorced and 11.7% were widowed. The mean duration of HIV was 12.1 ± 4.3 years. Only a small proportion of the participants (2.24%), had a history of smoking, and 32.9% of the participants had a history of alcohol consumption. The majority (47.4%) of the participants were farmers or livestock breeders. The mean age of the hypertensive subjects was 47.5 ± 7.6 years and normotensive individuals was 41.8 ± 6.9 years (p < 0.0001). The prevalence of hypertension was significantly different with marital status (p < 0.0001), duration of HIV (p = 0.007), WHO clinical stage (p = 0.02), diabetes mellitus (< 0.0001) and the BMI (p < 0.0001). There were no significant differences with smoking, alcohol consumption or gender (Table 1).

**Table 1:** Characteristics of HIV infected participants stratified by hypertension status.

Variables	Overall (n = 1250)	Hypertension		
		Yes (n = %)	No (n = %)	*p*-value
**Sex**				**0.38**
Male	230 (18.4)	35 (16.1)	195 (18.9)	
Female	1020 (81.6)	183 (83.9)	837 (81.1)	
**Mean age (years, mean ± SD)**	42.8 ± 7.4	47.5 ± 7.6	41.8 ± 6.9	** < 0.0001**
**Residence**				0.316
Rural	867 (69.3)	145 (66.5)	722 (69.9)	
Urban	383 (30.6)	73 (33.4)	310 (30.1)	
**Marital status**				** < 0.0001**
Single	29 (2.3)	4 (1.8)	25 (2.4)	
Married	820 (65.6)	110 (50.4)	710 (68.8)	
Divorced	147 (20.3)	68 (31.1)	186 (18.1)	
Widow	254 (11.7)	36 (16.5)	111 (10.7)	
**Duration of HIV (mean)**	12.1 ± 4.3	13.3 ± 4.2	11.75 ± 4.34	0.63
**Duration of HIV**				**0.007**
≤ 5 years	51 (4.1)	4 (1.3)	47 (4.5)	
5–9 years	342 (27.3)	46 (21.1)	296 (26.6)	
≥ 10 years	857 (68.5)	168 (77.1)	689 (66.7)	
**Duration group on ART**				0.193
≤ 5 years	58 (4.6)	6 (2.57)	52 (5.1)	
5–9 years	449 (35.9)	73 (33.4)	376 (36.4)	
≥ 10 years	743 (59.4)	139 (63.7)	604 (58.5)	
**WHO clinical stage**				**0.02**
Stage 1	209 (16.7)	41 (41.8)	168 (16.2)	
Stage 2	404 (32.3)	82 (37.6)	322 (31.2)	
Stage 3	572 (45.7)	91 (41.7)	481 (46.6)	
Stage 4	65 (5.2)	4 (1.8)	61 (5.9)	
**ART regim (TDF/3TC/DTG)**	1212 (96.9)	213 (97.7)	999 (96.8)	0.45
**HIV viral load (detectable)**	74 (5.9)	5 (2.2)	213 (97.7)	0.083
**Diabetes**	136 (10.8)	41 (18.8)	95 (9.2)	** < 0.0001**
**Current smokers**	28 (2.24)	0	28 (2.7)	**0.01**
**Alcohol use**	412 (32.9)	82 (37.6)	330 (31.9)	0.108
**Occupation**				
Farmer/breeder	593 (47.4)	103 (47.2)	490 (47.4)	0.779
Homemade/retired	336 (26.8)	56 (25.6)	280 (27.1)	
Jobless	191 (15.2)	38 (17.4)	153 (14.8)	
Employee/government employee	130 (10.4)	21 (9.6)	109 (10.5)	
**Body mass index**				** < 0.0001**
< 18.5	133 (10.6)	12 (5.5)	121 (11.7)	
18.5–24.9	649 (51.9)	98 (44.9)	551 (53.3)	
25.0–29.9	82 (37.6)	82 (37.6)	236 (25.4)	
≥ 30.0	26 (11.9)	26 (11.9)	97 (9.4)	

Significant values are in [bold].

**Figure 1:**
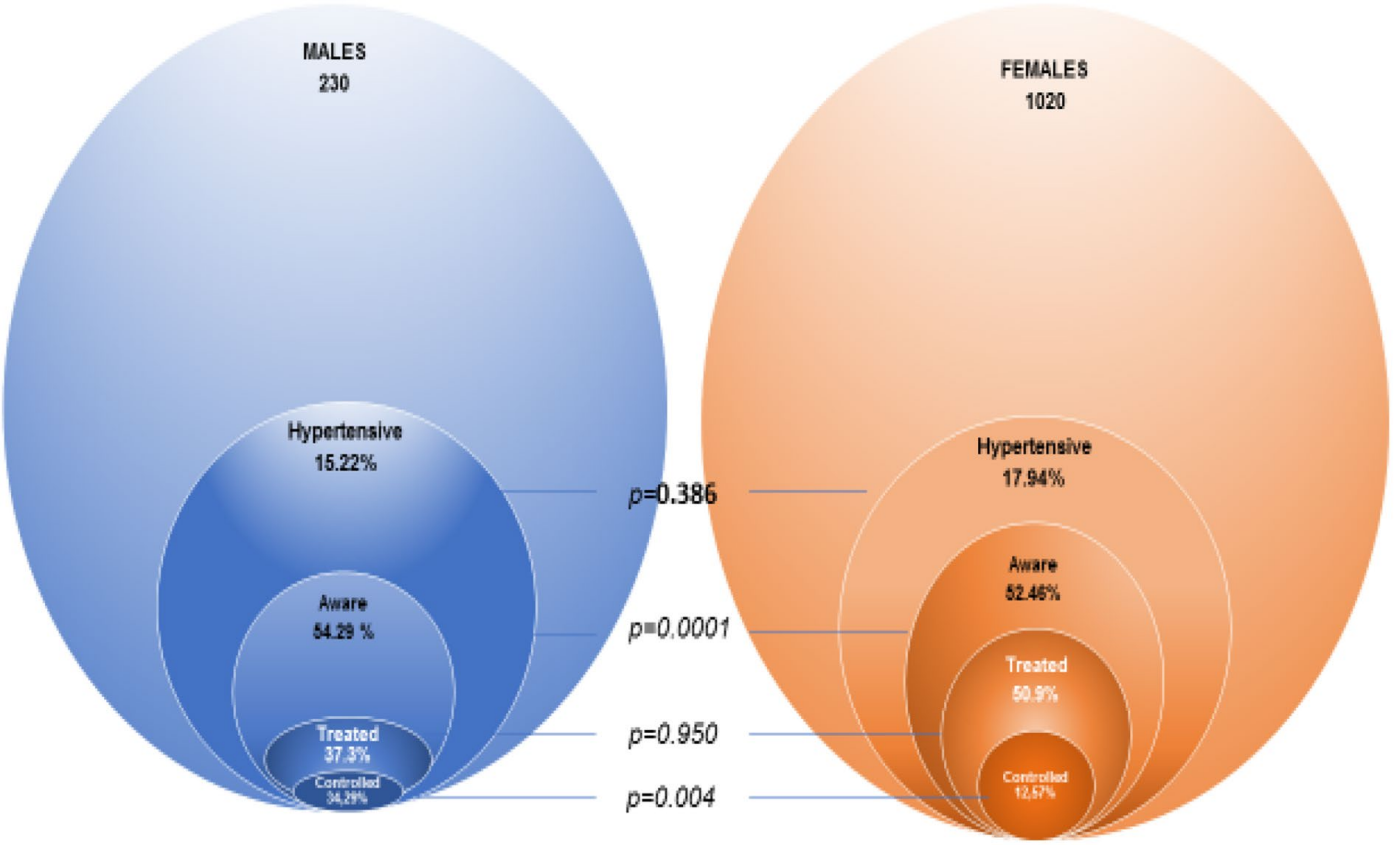
Hypertension awareness, treatment and control among 1250 HIV infected individuals on ART.

**Figure 2:**
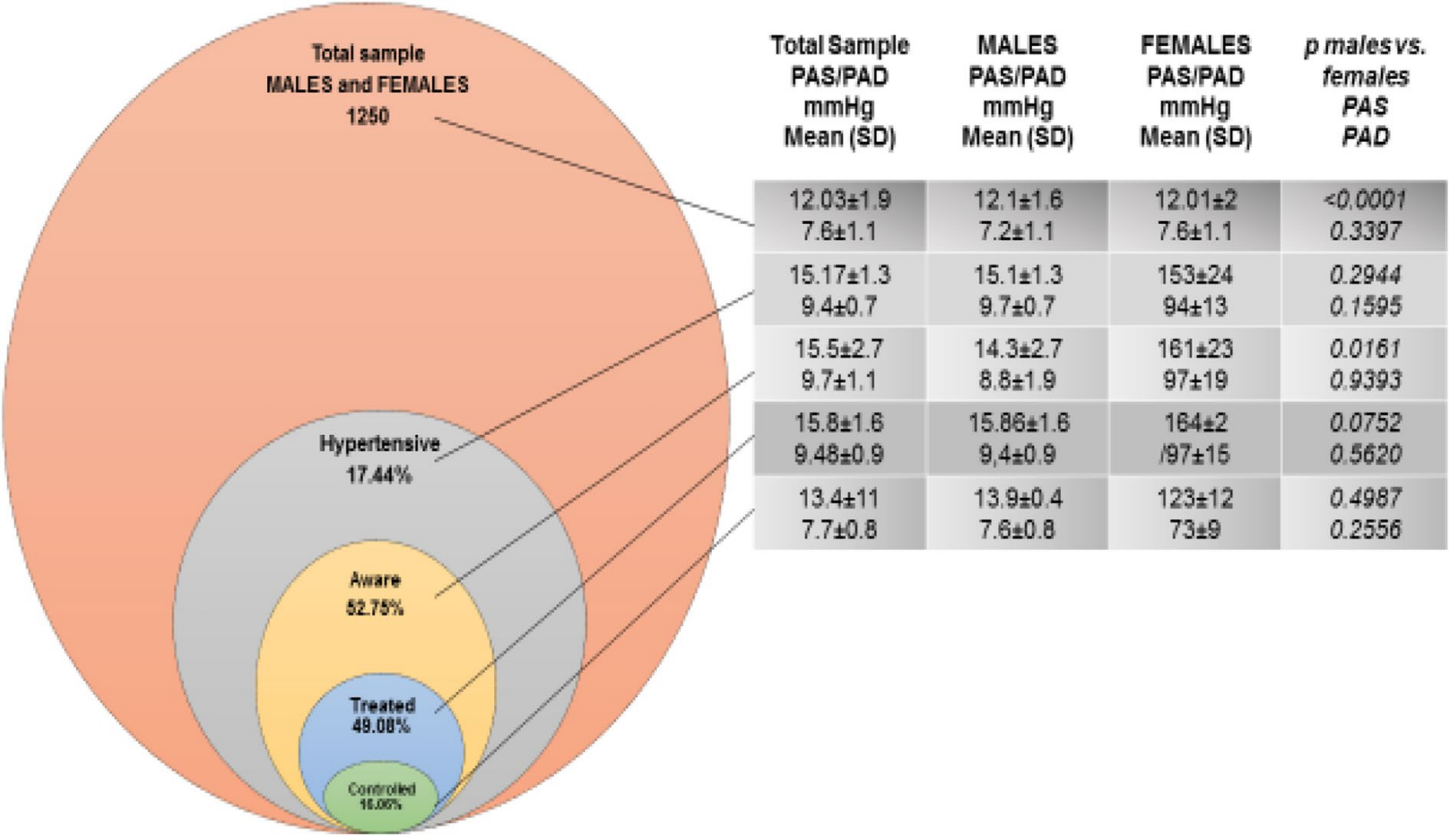
Prevalence and the proportion of treatment, controlled and awareness hypertension in study participants stratified to values of systolic and diastolic blood pressure in male and female.

The overall prevalence of hypertension was 17.4% (Fig. 2), with 15.2% men and 17.9% women (p = 0.38). Of the 218 participants with hypertension, 71 (47.2%) were not aware of their condition (Fig. 1). The mean SBP and DPP in newly identified HTN subjects was respectively 14.52 ± 0.6 cm Hg and 9.33 ± 0.5 cm Hg.

Among “older” HTN subjects, only 49.08% were on antihypertensive treatment and less than half (16.06%) met the criteria for blood pressure control (Fig. 2). There was no significant difference between men and women receiving antihypertensive treatment.

In univariate analyses (Table 2), age (OR = 1.1, 95% CI 1.08–1.13), age > 45 years (OR = 4.2, 95% CI 2.4–7.9) duration of HIV ≥ 10 years (OR = 2.8, 95% CI 1.5–3.4), BMI > 25 kg/m^2^ (OR = 3.1, 95% CI 1.6–5.9), BMI ≥ 30 kg/m^2^ (OR = 2.7, 95% CI 1.2–5.6) and WHO clinical stage III and IV of HIV infection.

**Table 2:** Factors associated with hypertension among HIV infected patients under ART in Burundi.

	Univariate analysis			Multivariate analysis		
	Odds ratio	CI 95%	*p*-value	Odds Ratio	CI 95%	*p*-value
**Age**	1.1	[1.08–1.13]	** < 0.0001**	1.1	[1.1–1.1]	** < 0.0001**
**Sex**						
Female	Ref					
Male	1.2	[0.8–1.8]	0.32			
**Age group**						
25–34	Ref					
35–44	1.3	[0.7–2.5]	0.304			
≥ 45	4.4	[2.4–7.9]	** < 0.0001**	4.2	[2.1–8.38]	** < 0.0001**
**Residence**						
Rural	Ref					
Urban	1.1	[0.85–1.60]	0.316			
**Marital status**						
Single	Ref					
Married	0.9	[0.3–2.8]	0.95			
Divorced	1.7	[0.7–6.8]	0.13	1.8	[0.1–1.1]	0.06
Widow	2	[0.6–6.2]	0.21	2.1	[0.2–2.4]	0.63
**Duration of HIV**						
≤ 5 years	ref					
5–9 years	1.8	[0.62–5.30]	0.26	5.39	[0.6–42.4]	0.109
≥ 10 years	2.8	[1–8.1]	** < 0.0001**	9.01	[1.0–76.2]	**0.043**
**Duration group on ART**	**0.1**	[0.9–1.1]	0.23	0.11	[0.7–1.2]	0.31
**WHO clinical stage**						
Stage 1	Ref					
Stage 2	1.1	[0.6–1.5]	0.84			
Stage 3	0.7	[0.5–1.1]	0.22	0.68	[0.4–1.1]	0.105
Stage 4	0.2	[0.0–0.7]	0.01	0.28	[0.01–0.8]	0.028
**Diabetes**	2.2	[1.5–3.4]	** < 0.0001**	2.05	[1.3–3.1]	**0.001**
**Alcohol use**	1.3	[0.9–1.7]	0.108	1.24	[0.8–1.7]	0.187
**Occupation**						
Farmer/breeder	Ref					
Homemade/retired	1.8	[0.7–1.7]	0.43			
Jobless	0.9	[0.5–1.5]	0.73			
Employee/government employe	0.9	[0.6–1.35]	0.78			
**Body mass index**						
< 18.5	Ref					
18.5–24.9	1.7	[0.9–3.3]	0.07	1.62	[0.8–3.1]	0.145
25–29.9	3.1	[1.6–5.9]	< 0.0001	3.05	[1.5–5.9]	**0.001**
≥ 30	2.7	[1.2–5.6]	0.008	2.4	[1.1–5.1]	**0.026**

Significant values are in [bold].

In multivariable analyses (Table 2), the factors associated with hypertension were age ≥ 45 years, duration of HIV ≥ 10 years, diabetes mellitus, BMI (25–29.9 kg/m^2^) and BMI ≥ 30 kg/m^2^.

## Discussion

To our knowledge, this is the largest study reporting the prevalence of hypertension in males and females treated as outpatients in urban and rural areas for HIV infection, and the first conducted in East Africa. The main finding and key message of our study is the very high prevalence of HTN in PLWH despite their young age (almost 20%), of whom almost half are unaware of their condition. The prevalence of 17.5% in our population is comparable to those reported in Nigeria^29^, Zimbabwe^30^, South Africa^31^ Zambia^32^ and Tanzania^33^, but lower than those reported in Uganda^34^, Cameroon^35^, Brazil^36^, China^17^,^18^ Ugandan rural study^37^ and Argentina^38^. Similarly, our findings are higher than those reported in Ethiopia^16,39,40^ and Kenya^41^.

The disparity in the prevalence of HTN among different sub-Saharan African countries may be related to several reasons, including genetic and socio-economic conditions, the duration and clinical stages of HIV, the types of ART therapy and the selected study population (e.g., hospitalized vs. outpatients).

The pathophysiology leading to cardiovascular disease in HIV infected patients is still controversial. Experiments with animals have suggested that a systemic inflammatory process and the activation of the adaptive immune systems would contribute to the development of HTN ^21,22,42^.

Long-term antiretroviral treatment, chronic inflammation and the immune activation associated with HIV infection, even if successfully treated, as well as the co-existence of some traditional cardiovascular risk factors expose subjects living with HIV to various morphological and metabolic disturbances, including features of the metabolic syndrome. This partly explains the increased risk of CV disease in the population living with HIV.

Several studies have demonstrated that possible risk factors for hypertension in the HIV-infected population are old age, male gender, family history of HTN, long duration of HIV infection, low CD4 count, high viral burden, diabetes, high body mass index and certain medications combined with ART^16,43,44^. In our study HTN appears to be related to age, being overweight and obesity, diabetes, duration of HIV and the combined antiretroviral therapy for more than 5 years.

Lubega and al. in their study on prevalence and factors associated with hypertension among people living with HIV/AIDS on antiretroviral therapy in Uganda found that hypertension was related to increasing age^34,44^. In our study, individuals older than 45 years were 4 times more likely to suffer from hypertension.

In our study, there was no relevant association between HTN and gender or smoking. This might be due to the general low percentage of current smokers in African countries^45^.

Compared to prevoius heterogeneous studies^46–48^ we found no difference in HTN prevalence between urban and rural areas.

The duration of ART and types of ART were reported, in particular protease inhibitors^32,38^. No association was found in our study. This may be due to the fact that Burundi has had a new protocol in place since July 2019, to rapidly advance to the next set of 2030 AIDS goals. 95% of people living with HIV were aware of their HIV status; 95% were on treatment; and 95% of people were on treatment with suppressed viral loads. The new WHO recommendations have switched dolutegravir to first-line treatment in settings where the resistance to non-nucleoside reverse transcriptase inhibitors (NNRTIs) is increasing^49^. Furthermore, the duration of ART and being HIV positive for over 10 years at least were associated with hypertension in our study.

In SSA, approximately one in three individuals with HIV have hypertension^20^. However, many HIV-positive people were unaware of their hypertension status. A study conducted in Tanzania showed barriers in integrating hypertension management for HIV control. The barriers were lack of hypertension knowledge, inefficient prescriptions, a lack of communication on related issues and hypertension care, in addition to high prices for healthcare in general, a lack of routine hypertension screening and follow-up^50^. In our study, almost 47.25% of HIV patients with hypertension were undiagnosed. In Tanzania and Brazil, HIV infected patients were not aware of their hypertension status, respectively 67.1% and 44.3%^36,51^.

In East African countries, the integration of HIV and NCD care is highly recommended in the policies of certain countries, namely Kenya, Rwanda, Tanzania and Uganda. Some countries, including Burundi, have placed particular emphasis on strengthening and developing health care infrastructures and have not yet established plans for effective integration of NCD and HIV care^52^.

The main limitation in our study is due to the cross-sectional design. Our study cannot chronologically determine the relationship between HTA and associated factors. In addition, due to lack of data in the Burundian population, our study does not compare HTN prevalence among HIV infected patients (17.4%) with HTN prevalence in the general population. The definition of HTN was limited to “one shot” measurements because ambulatory or home blood pressure measurements are unavailable in Burundi.

## Conclusion

This is the first study conducted on a large HIV infected population in Burundi, reporting a high prevalence of hypertension in HIV-infected patients. Findings revealed that age, being overweight, obesity, long duration of HIV infection and diabetes were consistently associated with hypertension. These results highlight the need to integrate hypertension management into routine HIV care in order to prevent adverse outcomes and to improve cardiovascular health in people living with HIV on antiretroviral therapy.

## Data Availability

The data that support the findings of this study are available from the corresponding author on reasonable request.
